# Expanding the social cure: a mixed-methods approach exploring the role of online group dance as support for young people (aged 16–24) living with anxiety

**DOI:** 10.3389/fpsyg.2023.1258967

**Published:** 2023-10-17

**Authors:** Saoirse Finn, Laura H. V. Wright, Hei Wan Mak, Emili Åström, Lucy Nicholls, Genevieve A. Dingle, Katey Warran

**Affiliations:** ^1^WHO Collaborating Centre for Arts & Health, Social Biobehavioural Research Group, Department of Behavioural Science & Health, Institute of Epidemiology and Public Health, University College London, London, United Kingdom; ^2^Childhood and Youth Studies Research Group, Institute for Community, Education, and Society, Moray House School of Education and Sport, The University of Edinburgh, Edinburgh, United Kingdom; ^3^Dance Base, Scotland’s National Centre for Dance, Edinburgh, United Kingdom; ^4^Music, Dance and Health Research Group, School of Psychology, The University of Queensland, Brisbane, QLD, Australia

**Keywords:** social cure, online, group dance, mental health, young people, loneliness, mixed-methods

## Abstract

There is an increased interest in whether online arts interventions support mental health and social connections. This study explored eight weeks of online group dance as support for young people (aged 16–24) living with anxiety. The applicability of the ‘social cure’ theoretical framework to the novel context of an online dance class was sought. The study utilised an embedded QUAL+quan design, incorporating participatory focus group discussions (*n* = 3 groups; *n* = 11 participants) and one-on-one interviews (*n* = 2 participants), creative reflections (*n* = 16 participants) and ethnographic fieldnotes, and a repeated measures design with surveys at three timepoints (week 1, *n* = 27; week 4, *n* = 18; week 8, *n* = 14). Thematic analysis identified two overarching themes demonstrating how the dance classes (i) provided the opportunity to co-construct a meaningful shared identity and (ii) supported holistic wellbeing. The quantitative findings supported this, suggesting lower anxiety, depression, and loneliness and higher wellbeing, self-esteem, self-efficacy, and group closeness. This study expands the social cure to its application to an online dance context for the first time.

## 1. Introduction

Mental health challenges often first occur during adolescence and, if left unresolved, can persist into adulthood (Kessler et al., 2007). Therefore, young people’s mental health is a global public health concern (Patel et al., 2007). Within the United Kingdom (UK), estimates suggest that one in six young people living in England (aged 17–22) report a mental health condition, with this rate increasing in recent years (NHS England, 2022). Symptoms of anxiety and depression are some of the most common experiences reported by young people (Lakasing and Mirza, 2020), and they are often positively correlated with loneliness, a subjective experience of psychological distress due to a perceived disconnect between individuals’ aspired and actual social connections (Hawkley and Cacioppo, 2010). Loneliness is also common in young people; in England, it is estimated that 26% of 16-24-year-olds feel lonely occasionally, 26% feel lonely sometimes, and 11% feel lonely often/always (UK Government, 2021). Factors contributing to anxiety, depression and loneliness interplay and include changes in social connections, life transitions, and mental and physical health (Patel et al., 2007; Lim et al., 2020).

During the COVID-19 pandemic, social interactions were severely affected, which had major effects on the mental health and loneliness of young people (Dingle et al., 2022), with research suggesting long-lasting impacts (Chadi et al., 2022). For example, compared to other groups, higher loneliness was found in young people and individuals with existing mental health conditions (Bu et al., 2020a,b; Varga et al., 2021), and young people were more likely to experience high anxiety and depression than other age groups during this time (Fancourt et al., 2021). This highlights the well-established interplay between loneliness and mental health. For example, poor mental health can lead to loneliness and being lonely can lead to worsened mental health (Hards et al., 2022). Therefore, it is important to consider effective ways to reduce loneliness and support mental health for young people, including support that can be accessed regardless of in-person restrictions.

In a recent conceptual framework of interventions to support mental health and loneliness in young people, three activity content strategies were proposed (Pearce et al., 2021). Intrapersonal strategies included therapy (e.g., cognitive behavioural therapy (CBT)) as well as self-help activities (e.g., exercise or listening to music) eliciting positive self-attitude mechanisms. Interpersonal strategies included social skills content that facilitates mechanisms relating to social confidence. Finally, social strategies included activity content that increases social contact (including online groups), which fosters mechanisms such as a sense of community (Pearce et al., 2021). Importantly, social group activities have been found to have similar effect sizes as therapy-based interventions for mild to moderately depressed participants (Dingle et al., 2021). However, further research is needed to explore the complexity of the interrelationship between individual-level and group-level factors in the context of social group activities for supporting mental health and loneliness in young people.

One approach that acknowledges these complexities is the ‘social cure’. The social cure is commonly viewed as a theory from social psychology, but it is rooted in sociological theory tracing back to Durkheim (1897, 1912). Durkheim first proposed that integration into societal groups is protective (Durkheim, 1897) and that group belonging can foster collective effervescence – a feeling of collective emotion that meaningfully binds group members together (Durkheim, 1912). Building on this, sociologists and social psychologists have sought to further unpack the mechanisms of how meaningful social identities are created (Collins, 2004) and how these social identities may support different dimensions of health and wellbeing (Jetten et al., 2012; Kawachi and Berkman, 2014).

Two important theories are *social identity theory* and *self-categorisation theory,* which have been combined to construct a social identity approach to health: ‘the social cure approach’ (Jetten et al., 2012; Haslam et al., 2018). The social cure theorises a relationship between group-level factors (e.g., feelings of social identity) and improved individual-level factors (e.g., mental health) (Haslam et al., 2018). Within this theoretical landscape, social identity refers to an internalised group membership that can contribute to a person’s sense of ‘who they are’ in a given context, with an individual’s self-identity viewed in terms of meaningful relations with others in group contexts (Haslam et al., 2018). This builds on Tajfel’s (1972) assertion that belonging to certain social groups may have emotional or value significance that contributes to the construction of social identity (Tajfel, 1972; Haslam et al., 2018). Meaning is therefore a central concept within the social cure approach, as when individuals experience group-based interactions as purposeful, benefits for health are seen (Haslam et al., 2018). These benefits are theorised as psychological resources that support mental health by providing a sense of collective meaning, purpose, personal agency, belonging, social support, and efficacy (Greenaway et al., 2016; Haslam et al., 2022). In sum, the social cure approach theorises that social identity enables psychologically meaningful group memberships that support mental health and protect against loneliness through the provision of important psychological resources (Haslam et al., 2022; Hayes et al., 2022). One avenue to explore the social cure approach is via identification with arts groups.

Arts activities are complex interventions as they involve multiple interacting components (Warran et al., 2022), which prompt mechanisms that interplay with mental and physical health (i.e., through social, psychological, behavioural, and biological processes) (Fancourt et al., 2021). Engagement with arts activities has been found to have wide-ranging health benefits across the life course (Fancourt and Finn, 2019), and there is increasing interest in asset-based and community-based approaches (e.g., Social Prescribing) to support health and wellbeing (Morse et al., 2022).

There is evidence that in-person group arts engagement can support young people’s mental health. For example, engaging in arts activities has been found to support mental health and resilience through building self-confidence, self-esteem (Bungay and Vella-Burrows, 2013; Zarobe and Bungay, 2017), and belonging (Zarobe and Bungay, 2017). Music activities have been found to support depression, anxiety, and stress (Daykin et al., 2018). Group dance has been found to support wellbeing by alleviating feelings of depression and distress (Mansfield et al., 2018) and supporting self-concept, self-esteem, self-efficacy, self-expression, and self-trust (Schwender et al., 2018). However, there has been less research on group arts engagement supporting loneliness in younger populations (Bone et al., 2022). Several studies have applied the social cure approach to their methodological design to examine the role of arts activities on health outcomes in different populations (Williams et al., 2019, 2020; Dingle et al., 2020; Forbes, 2021). However, no studies have applied the social cure approach to arts activities specifically for young people’s mental health or loneliness, or for dance activities. Additionally, no studies have applied the social cure approach to an online arts intervention, which came to the fore as a priority research gap during the onset of the COVID-19 pandemic.

In response to the COVID-19 pandemic, there was a move from in-person to online arts engagement (Bradbury et al., 2021), with research finding that engaging in arts activities during the pandemic supported mental health and wellbeing across different age groups (Cabedo-Mas et al., 2020; Bu et al., 2021; Kiernan et al., 2021; Nie et al., 2021). There has been some research on the role of online group dance as a support for mental health and loneliness. For example, after a single session for adults, increases in positive affect, self-esteem, social connectedness, and community connectedness were found, as well as decreases in negative affect and depressive symptoms (Humphries et al., 2023). A 12-session dance program for intimate partner violence survivors’ mental health found some evidence of a reduction in distress (Özümerzifon et al., 2022). Whereas a 5–10 min activity of online physical movement (including ballroom dancing and endurance exercises) or creative writing before classes found no effects on depression, mood, stress, or quality of life across a semester (Marschin and Herbert, 2021). Overall, the literature on online dance is scarce, and more is needed to explore how it may support young people’s mental health and loneliness.

To address the need for supportive interventions for young people’s mental health and loneliness, which are available regardless of in-person social restrictions, this study aimed to explore if and how online group dance classes can support young people living with anxiety, applying the social cure as a theoretical framework. Our research questions asked:

1. Do young people living with anxiety experience perceived changes to their mental health and loneliness after participation in 8-weeks of online group dance classes?
2. Do participants attribute any of these perceived changes to the co-construction of a group identity created through these shared online experiences?
3. What factors (e.g., the online context) play a role in any perceived changes to their mental health and loneliness?

## 2. Materials and methods

### 2.1. QUAL+quan

To explore the research questions in-depth and to tackle complexity, different perspectives were explored in a mixed-methods design using a concurrent embedded approach, whereby the qualitative methodology was the driver of the study, and the quantitative component was nested within it (QUAL + quan) (Creswell and Creswell, 2017). The qualitative component focused on theoretical innovation of the social cure and understanding subjective experiences, while the quantitative component measured potential changes to mental health, wellbeing, loneliness, and group processes using a repeated measures design with three assessments over eight weeks (after classes 1, 4, and 8).

Taking a participatory co-production approach was also central to our methodology: we worked in partnership with an Intergenerational Advisory Committee (IAC) (noted in the Acknowledgements) comprised of youth advisors (aged 16–24) with lived experience of anxiety and/or an interest in dance and health research, senior academics, and Dance Base (our charity partner). Reporting on our participatory processes will be published elsewhere (Wright et al., n.d.).

### 2.2. Participant recruitment

Eligibility criteria included young people who: (i) were aged 16–24 and living in the UK; (ii) were living with anxiety (determined by a score of ≥5 on the Generalized Anxiety Disorder Assessment Scale (GAD-7)); (iii) had access to a computer or smartphone to join the classes; and (iv) could give informed consent. This was assessed via a publicly available screening form on Microsoft Forms. Additionally, access needs were asked about to support inclusion. There were no financial incentives to be involved, but the dance classes were provided free of charge.

We sought to recruit a diverse sample across age and ethnicity. However, due to recruitment challenges (e.g., low take-up and lack of resources), our strategy fell closer to a form of convenience sampling, details of which are reported in the Supplementary material. Overall, 86 individuals were screened, 69 were eligible and contacted via email and sent participant information sheets and consent forms, 35 returned consent forms and 27 enrolled in the first dance class. Those who were not eligible were also emailed information about other free arts and mental health resources they may wish to access.

### 2.3. Online dance classes

The online dance classes were delivered by Dance Base, an organisation that began hosting classes online in response to COVID-19. Dance Base coordinated the dance classes, and the research team coordinated data collection with the project called Dance/Connect for recruitment purposes. Sixteen online dance classes were delivered in two blocks and ran weekly over eight weeks every Thursday evening via password-protected Zoom meetings between September 30th to November 18th (block one) and October 28th to December 6th (block two) 2021. The same dance practitioner facilitated the classes each week, except for two classes when they were unavailable. A moderator also joined every class to provide practical and technical support. The genre of the classes was decided in consultation with the IAC as ‘beginner contemporary’. The dance practitioner designed the content with feedback from the IAC, made the playlists (including pop, country, instrumental and festive music), and facilitated the dance and creative elements. Safeguarding and accessibility were priorities, with Dance Base, the research team, and the IAC considering these factors in the design and delivery of the classes. Each class was 90 min and incorporated ice-breakers, improvisation, imagery tasks, and the main dance movement exercises. The structure of the dance classes is presented in Supplementary Table S1. Due to these activities’ complexity and dynamic nature, there were minor variations across the weeks.

### 2.4. Demographics, health conditions, and arts behaviours

In week 1, questions on sociodemographics, health and arts behaviours were asked. This included age, gender, ethnicity, and parent’s/carer’s highest educational level (to assess socioeconomic background). For gender and ethnicity, participants were given the option to choose a listed term, not to disclose, or state their own term in an open-ended format. The presence of a physical health condition, mental health condition, or disability was also asked if participants wanted to disclose. Arts behaviours were asked about before the COVID-19 pandemic and during/since the pandemic. This was to assess the level of arts engagement of participants who participated in the study and how COVID-19 may have impacted general arts behaviours. Please refer to the Supplementary material for more information on these measures.

### 2.5. QUAL design and analysis

Participants were invited to submit a creative journal reflection after week 4 of the classes and to participate in an online focus group in week 8, before their final class. There were three separate focus groups, and due to the availability of participants, two participants took part in individual one-on-one interviews instead. Two core research team member with social science backgrounds (KW, LW) and a youth advisory member who received focus group training facilitated focus groups and interviews. All participants were invited to participate in the creative reflections and focus group/interviews, even those who had not attended throughout (but had at least joined in week 1). In addition, the dance practitioner and moderator engaged in ethnographic reflections after each class (see Supplementary material for this template).

The focus groups and interviews utilised an arts-based method known as ‘river journey’, which involved inviting the young people to reflect on their experiences of participating in the online classes by visually mapping out how they felt at the start, middle and end of the eight weeks (Lee et al., 2022). They were invited to draw a river using colored pens and pencils across a blank page, writing keywords and images that reflected their significant experiences, strengths, and challenges. This technique was employed to provide an opportunity for creative expression and reflection (McNiff and Whithead, 2012). Participants were asked to share their reflections on engaging in the river journey exercise and hold their images up for other participants to see if they would like to. The facilitators also asked questions on relevant aspects of the drawings to explore the research aims. Following engagement with the river journey exercise, a semi-structured interview guide (see Supplementary material) was used to discuss topics relating to group membership, social support, mental health, wellbeing, barriers and enablers to engaging with the dance classes, and subjective experiences.

Focus group and interview discussions were transcribed verbatim by a secure transcription agency and analysed using reflexive thematic analysis in NVivo v.12, along with the creative journal reflections (text but not images) and ethnographic reflections. The river journey drawings themselves were not analysed, but the discussions of the exercise were. We drew upon Braun and Clarke (2022), which involved familiarising ourselves with the material, generating initial codes, identifying and reviewing themes, and defining and naming themes (Braun and Clarke, 2022). We initially took a deductive approach to analysis, drawing on a framework created out of key themes relating to the social cure (see Supplementary material). However, this was complemented by open, inductive coding and memo-writing when new or interesting findings emerged. Initial analysis was completed by KW with supporting analysis and second coding from LN, with themes discussed and refined in a series of discussions between KW, LN, and LW. The discussions also included reflection on researcher positionality. In this article, participant names are pseudonymised to protect the anonymity of the participants.

### 2.6. Quan design and statistical analyses

#### 2.6.1. Design and data collection

A one-group repeated measures design with three assessments was used, assessing changes within subjects across eight weeks. Surveys were emailed to participants at three timepoints: after the dance classes in week 1, week 4, and week 8. The questionnaires were completed using Microsoft Forms and took 10–15 minutes on average to complete.

#### 2.6.2. Measures

##### 2.6.2.1. Mental health, wellbeing, and loneliness

Across all three timepoints, mental health, wellbeing, and loneliness were measured (see Supplementary material for further details of these measures).

*Anxiety* was measured using the GAD-7, a validated tool for screening anxiety and its severity in clinical and research settings (Spitzer et al., 2006). It has seven items measured on a 0–3 point scale ranging from “*Not at all*” to “*Nearly every day*,” with higher scores (ranging from 0–21) indicating higher anxiety and cut-off points to indicate different levels of anxiety (<5 for no anxiety, 5–10 for mild, 10–15 for moderate, and >15 for severe anxiety) (Spitzer et al., 2006).

*Loneliness* was measured using the three-item UCLA-Loneliness Scale (UCLA-3), a validated tool for use in surveys (Hughes et al., 2004). Items are measured on a 1–3 point scale ranging from “*Hardly ever*” to “*Often*,” with higher scores (ranging from 3–9) indicating higher loneliness (Hughes et al., 2004).

*Depression* was measured using the two-item Patient Health Questionnaire (PHQ-2), a validated tool for depression screening (Kroenke et al., 2003). Items are measured on a 0–3 point scale ranging from “*Not at all”* to *“Nearly every day,”* with higher scores (ranging from 0–6) indicating higher depression (Kroenke et al., 2003).

*Wellbeing* was measured using the short version of the Warwick-Edinburgh Mental Wellbeing Scale (SWEMWBS), validated for use in the general population (Stewart-Brown et al., 2009). It has seven items measured on a 1–5 point scale ranging from *“None of the time”* to *“All of the time,”* with higher scores (ranging from 7–35) indicating higher wellbeing (Stewart-Brown et al., 2009).

*Self-esteem* was measured using the one-item self-esteem scale (Robins et al., 2001). Participants were asked to rate whether they had high self-esteem on a seven-point Likert scale (i.e., *“1 = Not very true of me”* to *“7 = Very true of me”*) (Robins et al., 2001).

*Self-efficacy* was measured using the six-item General Self-Efficacy Scale (GSE-6) (Romppel et al., 2013). Participants were asked to describe their opinions (e.g., *“Thanks to my resourcefulness, I know how to handle unforeseen situations”*) on a rating scale of 1–4 (i.e., *“Not at all true”* to *“Exactly true”*). Scores were summed (ranging from 6–24), with higher scores indicating higher self-efficacy (Romppel et al., 2013).

##### 2.6.2.2. Group processes

Collective efficacy, group support and group identity were asked from week 4, as these measures were deemed less relevant to participants at week 1 and which also reduced the time taken to complete the first survey (where demographic information was taken). However, trust in the teacher and group closeness were asked from week 1 (see Supplementary material for further details of these measures).

*Trust in the teacher* was measured by a single item, asking participants to rate their trust in the dance class teacher (i.e., *“I have trust in the teacher”*) on a seven-point Likert scale (i.e., *“1 = Not at all”* to *“7 = Very much”*).

*Group closeness* was measured using the Inclusion of Other in Self Scale (IoS scale), a graphical representation of closeness (Aron et al., 1992). This was adapted to ask participants to describe their relationship with the dance group and was measured on a seven-point Likert scale, with higher scores indicating greater group closeness (Aron et al., 1992).

*Group support* was measured using a one-item question that was created for this study and included the statement *“I feel supported by the dance group*,” rated on a seven-point Likert scale (i.e., *“1 = Strongly disagree”* to *“7 = Strongly agree”*).

*Group identity* was measured using the four-item social identity scale (FISI) (Doosje et al., 1995). It was adapted to include statements relating to dance (e.g., *“I identify with the dance group”*) rated on a seven-point Likert scale (i.e., *“1 = Strongly disagree”* to *“7 = Strongly agree”*). The items were averaged to yield a score from 1 to 7, with scores above 4 (the neutral point) indicating group identification.

*Collective efficacy* was measured using a one-item scale utilised in numerous studies (Cruwys et al., 2020). It was adapted to ask, *“Together with other dance group members, we have been able to accomplish new dance skills and learn new choreography,”* rated on a seven-point Likert scale (i.e., *“1 = Strongly disagree”* to *“7 = Strongly agree”*).

#### 2.6.3. Statistical analyses

One-way repeated measures analysis of variance tests (RM ANOVA) were run to test whether there was evidence of differences in the means of measures across the timepoints using complete case analysis (i.e., *n* = 14). If RM ANOVA indicated differences, post-hoc tests were run to explore this further. This was done via estimation of predicted means for each timepoint, and pairwise comparisons of these means to assess between which timepoints differences were being observed and the magnitude of the differences by reporting contrasts. Sensitivity analyses were also conducted with the full sample (different sample sizes across timepoints) to compare with the complete case findings and are presented in the Supplementary material. All analyses were conducted in STATA release 17 (STATA Corp LP, USA).

## 3. Results

### 3.1. Participant demographics

The participant’s data in both dance class blocks were combined across weeks. Table 1 presents the participant demographics, and Table 2 presents their arts behaviours; these data were collected in the week 1 (*n* = 27) survey. However, they are also presented for participants who completed week 4 (*n* = 18) and week 8 (*n* = 14) surveys, as well as focus groups (*n* = 13).

**Table 1:** Demographics for participants who completed surveys and focus groups/interviews.

	Week 1 (*n* = 27)	Week 4 (*n* = 18)	Week 8 (*n* = 14)	Focus groups/interviews (*n* = 13)
	**Mean (SD)**			
**Age (years)**	21.0 (2.1)	20.8 (2.2)	20.7 (2.2)	20.4 (2.4)
	**Percentage % (*n*)**			
**Gender**				
Female	85.2 (23)	83.3 (15)	92.9 (13)	92.3 (12)
Gender non-conforming	3.7 (1)	5.6 (1)		
Non-binary and transgender	3.7 (1)			
Gender queer	3.7 (1)	5.6 (1)	7.1 (1)	7.7 (1)
Gender non-conforming and non-binary	3.7 (1)	5.6 (1)		
**Ethnicity**				^*^
White British	37.0 (10)	50.0 (9)	42.8 (6)	
White Irish	3.7 (1)			
Other White background	33.3 (9)	38.8 (7)	42.8 (6)	
Mixed/Multiple ethnic background	7.4 (2)	5.6 (1)	7.2 (1)	
Pakistani	3.7 (1)	5.6 (1)	7.2 (1)	
Chinese	14.8 (4)			
**Parental education**				
Parent/carer 1				
Do not know or prefer not to say	7.4 (2)	11.1 (2)	14.3 (2)	7.7 (1)
School and college qualifications	11.1 (3)	5.6 (1)		7.7 (1)
Undergraduate degree or above	81.5 (22)	83.3 (15)	85.7 (12)	84.6 (11)
Parent/carer 2				
Do not know or prefer not to say	22.2 (6)	33.3 (6)	42.8 (6)	28.5 (5)
School and college qualifications	25.9 (7)	27.8 (5)	21.4 (3)	23.0 (3)
Undergraduate degree or above	51.9 (14)	38.9 (7)	35.7 (5)	28.5 (5)
**Lived experience of mental health**				
Yes	66.7 (18)	66.7 (12)	64.3 (9)	61.5 (8)
No	18.5 (5)	22.2 (4)	28.6 (4)	23.1 (3)
Prefer not to say	14.8 (4)	11.1 (2)	7.1 (1)	15.4 (2)
**Lived experience of physical health**				
Yes	18.5 (5)	16.7 (3)	85.7 (2)	15.4 (2)
No	77.8 (21)	83.3 (15)	14.3 (12)	76.9 (10)
Prefer not to say	3.7 (1)			7.7 (1)
**Lived experience of disability**				
Yes	14.8 (4)	16.7 (3)	14.3 (2)	15.4 (2)
No	85.2 (23)	83.3 (15)	85.7 (12)	84.6 (11)

SD = standard deviation, *n* = number of participants. ^*^Ethnicity data is not reported for the qualitative data to protect the anonymity of participants.

**Table 2:** Arts behaviours for participants who completed the surveys.

	Week 1 (*n* = 27)	Week 4 (*n* = 18)	Week 8 (*n* = 14)
	**Percentage % (*n*)**		
**Before COVID-19, how often would you say you engaged in arts/creative activities generally?**			
Less than once a year	3.7 (1)	5.6 (1)	7.1 (1)
A few times a year	25.9 (7)	16.7 (3)	21.4 (3)
At least once a month	29.6 (8)	33.3 (6)	28.6 (4)
At least once a week	40.7 (11)	44.4 (8)	42.9 (6)
**Thinking about your engagement levels throughout the COVID-19 pandemic, has there been any change in your levels of engagement with arts/creative activities?**			
I engage less than before the pandemic	37.0 (10)	33.3 (6)	42.8 (6)
There has been no change	33.3 (9)	33.3 (6)	28.6 (4)
I engage more than before the pandemic	29.6 (8)	33.3 (6)	28.6 (4)
**Since the COVID-19 pandemic started, how has your online engagement with these arts/creative activities changed?**			
I engage less	14.8 (4)	5.6 (1)	7.1 (1)
There has been no change	37.0 (10)	44.4 (8)	35.7 (5)
I engage more	48.2 (13)	50.0 (9)	57.1 (8)

*n* = number of participants.

At week 1, the mean age of participants was 21 years old (SD = 2.1, range: 17–24). The majority of the participants identified as female (*n* = 24), were of White ethnic background (*n* = 20), reported lived experience of mental health (*n* = 18), did not report lived experience of physical health (*n* = 21) or disability (*n* = 23), and 67% had at least one parent/carer with an undergraduate degree or above. A majority engaged frequently with arts and creative activities before the COVID-19 pandemic (at least once a month: *n* = 8, at least once a week: *n* = 11). The differences in engagement levels throughout COVID-19 were fairly equal across engaging less now, no change and engaging more now. However, slightly more participants appeared to engage more with online activities in response to COVID-19 (*n* = 13).

### 3.2. QUAL findings

Two overarching themes and nine subthemes were constructed through the analysis procedure (see Table 3). The themes show how the dance classes provided an opportunity for many who participated to co-construct a meaningful shared identity that represented inclusivity and supported holistic wellbeing.

**Table 3:** Description of overarching themes and subthemes derived from the analysis procedure.

Theme	Subtheme	Description
**Co-constructing a shared identity**	1.1 Alternatives to belonging	Participants rejected the language of ‘belonging’ in relation to how they felt about the group, preferring “team” (Rosie, FG1), “nurturing environment” (Imani, FG3) and “safe space” (Bridget, FG3)
	1.2 Symbols of shared identity	Institutional affiliation (e.g., to Dance Base and UCL), pictures created in the classes, and the language of “dance terminology” (Ethnographic reflection) acted as meaningful symbols representing participants association with the dance group
	1.3 Going through shared experiences	A sense of shared experience (“on the same frequency” Flora, FG2) was created among dance class participants through shared mental health experiences and dancing together
	1.4 The meaning of the group	The group was important to participants seen through their “love” (Imani, FG3) of dancing and their willingness to prioritise attendance in view of other commitments
**Improved holistic wellbeing**	2.1 Psychological wellbeing	The classes contributed to improved confidence, self-care, and mood (“good energy” Lily, Interview) and distracted from anxiety and stress (“let go of worries” Sophie, Creative reflection)
	2.2 Physical and bodily wellbeing	The classes supported with the physical aspects of having anxiety (e.g., releasing tension) and increased physical activity (e.g., exercising)
	2.3 Social wellbeing	Participants felt a “fondness” (Bridget, FG3) for others in the group, which was perceived as “like a family” (Rosie, FG1), which provided social support
	2.4 Wider behavioural change	The classes supported with building general confidence and with “academic participation” (Kiera, Creative reflection), impacting routines in a positive way
	2.5 Structure and content of the online classes	The content of the dance exercises was viewed as inclusive, with the weekly nature of the classes adding “structure” (Grace, FG2) to participants’ lives

#### 3.2.1. Theme one: co-constructing a shared identity

The first theme explores the *co-construction of a shared identity* through engagement with the classes for many who participated in the group. Participants recounted their experiences of navigating what the shared identity of the group was, and collectively, they negotiated the language that they felt most appropriately represented their sense of affiliation.

##### 3.2.1.1. Alternatives to belonging (subtheme 1.1)

In one focus group (FG), this negotiation entailed a rejection of the language of ‘belonging to a group’ (the dominant language of the social cure) in favor of *alternatives to belonging* such as ‘team’, ‘environment’ and ‘safe space’. This rejection was founded on a feeling that ‘belonging’ inherently entails that some people will not belong, with most of our participants experiencing the classes as inclusive:

*“I don’t think the first thing I felt was belonging, but it was more, like, you didn’t need to belong to it because it [the dance class] felt really open so anyone could belong, so it wasn’t necessarily that you had to fit in… I think it was just very welcoming of everyone and there wasn’t judgement… I felt comfortable to kind of dance, or move however.” (Priya, FG2).*

*“…yeah, belonging, definitely I feel like that was wrong, because the idea of belonging is very much for me, like, a subordinance to a group in a sense of also ‘fitting in’, like, it’s constrictive.” (Molly, FG2).*

*“…it’s just the way the word ‘belonging’ is used usually, in the context of ‘you do not belong’; it kind of suggests that you need like the community’s approval or something to be considered a ‘member’, like, someone who’s truly ‘belonging’. And in that sense, it’s very exclusive and I, that’s not what this was, in like a good way.” (Flora, FG2).*

*“I’d kinda um describe it as like a kind of encouraging and nurturing environment.” (Imani, FG3).*

While the language of ‘team’ sometimes has connotations of competition, for our participants, it was the collaborative and inclusive aspects of a team that resonated (*“I think maybe just like a team,” Rosie, FG1*). Another preferred alternative was a ‘safe space’ (“*terming it as a space or a safe space,” Bridget, FG3*), which was viewed as more suitable language for an online environment (“*online you sort of feel like part of that space,” Bridget, FG3*).

Thus, it was important for many of our participants not to use the language of ‘belonging’ as they did not see the space as exclusionary or value spaces that fostered/allowed for exclusion. Additionally, they did not see themselves as exclusive ‘members’ of a ‘membership group’. Instead, they viewed the class as breaking down barriers and an inclusive experience. However, one of our participants disagreed, describing feelings of exclusion due to their ethnicity:

*“… both the people who are teaching were white, and everyone else in the class was white. So, if you only have one person of colour and they already feel uncomfortable, so they’re not willing to speak up about it, then you’re just not going to get the right experience because there’s just not enough diversity within the group as a whole.” (Lily, Interview).*

While many of our participants felt that their experiences transcended the idea of ‘group’ creation or ‘belonging’, this participant’s experiences highlight that boundaries were present and that more needs to be done to improve diversity and inclusion of ethnic minority groups in dance class participation.

##### 3.2.1.2. Symbols of shared identity (subtheme 1.2)

Interconnected to subtheme 1.1, the next subtheme explores *symbols of the group’s co-constructed shared identity*, referring to symbols that represented the shared experience of the dance classes. There were different dimensions to this, with participants bonding over various aspects of the classes. This was most prominent in relation to institutional affiliation. Several participants felt affiliated with University College London (UCL) ahead of joining the classes, meaning that it was this aspect of the classes that they felt most connected to (*“It’s a study run by UCL. That’s where the connection comes in,” Lily, Interview; “I also said I was doing a research project for UCL,” Rosie, FG1*).

Other participants felt that they had a closer affiliation with Dance Base. For some, this was because they were based in Edinburgh (“*I’m Edinburgh-based and I know Dance Base,” Olivia, FG1*), and, for others, they became closer to the organisation during their experiences of Dance/Connect (*“I think just because like a ‘base’ feels like home almost it’s a solid foundation,” Zara, Interview*).

One participant described how they previously thought about joining classes at Dance Base but “*never ended up engaging” (Molly, FG2)*, with the Dance/Connect project spurring them on to consider joining one now:

*“The reason why I might start one [a dance class at Dance Base], I’ll probably start one, is this experience has taken me closer to Dance Base*.” *(Molly, FG2).*

Many participants described their sense of connection to the dance practitioner and moderator as mechanisms underpinning their increased sense of affiliation to Dance Base. One even shared that they would turn their camera on just so the dance practitioner *“did not feel on her own” (Rosie, FG1)*. However, for one participant, while they felt some connection to Dance Base, this was not strong enough to support feelings of bonding (*“in terms of do I feel a bond to them, probably not*,” *Molly, FG2*).

Other participants reported feelings of connection emerging for Dance Base but not as strong as other institutional affiliations, such as to UCL:

*“But I wouldn’t necessarily say that translates into me feeling as connected to Dance Base as I do to UCL. Obviously, because I go to UCL as a university, I think the like, 99 per cent of the connection is always going to lie with them.” (Lily, Interview).*

Very few participants felt any connection to the language of ‘Dance/Connect’, choosing to refer to the classes in view of either UCL and/or Dance Base based on their prior connections. Other symbols that emerged were the pictures drawn in the classes and kept, functioning as representations of the group (“*I would have them [the drawings] on my desk in my room,” Molly, FG2*), and a gestural hand task that the dance practitioner used as an ice-breaker activity which represented fun and enjoyment *(Grace, Creative reflection, and Ethnographic reflection)*. Learning to dance was also itself a symbol in the form of a new shared language, with participants learning the *“meaning of dance terminology” (Ethnographic reflection)*.

##### 3.2.1.3. Going through shared experiences (subtheme 1.3)

Despite different institutional connections that were operating across dance class participants, there was still a strong sense of *shared experience* created through a shared sense of everyone having anxiety and engaging in the dance classes together:

*“It was also nice to have the breakout rooms and to see that people were actually feeling the same thing. Or some people felt that they were developing the same thing and they were enjoying the same thing as you.” (Zara, Interview)*.

“…*having this shared, similar, experience… It’s not a ‘*dance *group, dance group’ it’s a dance group of other people who think a little bit like me.” (Flora, FG2)*.

Moreover, the individual who reported feeling excluded from the group due to their ethnicity also reflected a feeling of shared experience through the shared demographics of all being *“a similar age and… most of us being women” (Lily, Interview)*.

This sense of shared experience was viewed as taking a *“risk”* together to “*share our selves, our minds, with each other”* (*Molly, Creative reflection*), there was a feeling of being *“on the same frequency, without anyone trying to put us on that frequency” (Flora, FG2)* and leaving the classes *“wanting to do the same thing or have the same, like a similar thing to do” (Zara, Interview)*.

##### 3.2.1.4. The meaning of the group (subtheme 1.4)

Bringing together all the subthemes in this section, the last subtheme demonstrates how the group’s co-constructed identity and shared experiences created *a meaningful dance group*. One participant felt that the classes were important because they experienced a sense of ‘liberty’ in joining them, suggesting the classes represented freedom for them. Another described meaning as coming from engaging in music and dance that is *“borrow[ed] from the works of others in order to find my own definition of peace” (Lily, Creative reflection)*. This was reinforced by others who felt that engaging in dance enhanced their lives:

*“It's a reminder of how I feel when I'm dancing and that I want to, you know, keep it in the life and enhance it.” (Ana, FG1)*.

*“This opportunity showed me that I, like, loved it (dancing).” (Imani, FG3)*.

The meaning of the group was also communicated through prioritizing the classes (*“always prioritising regardless of what was going on in my week”*), as well as giving the dance group meaning beyond the allotted time of the classes themselves *(“I think it [the dance group] has started leaking into my identity and my life as a whole”) (Mia, FG2)*.

In summary of theme one, participants co-constructed a shared sense of group identity through weekly dance participation, rejecting the language of ‘belonging’ and instead focusing on an inclusive space of shared experience where participants could derive meaning from dancing together.

#### 3.2.2. Theme two: improved holistic wellbeing

The next theme describes how meaning connected to the dance classes also supported with *improved holistic wellbeing*. Participants experienced interconnected and shared ‘bodily’ and ‘mental’ wellbeing that influenced both in-the-moment wellbeing and wider positive behavioural change. Their time for dancing was viewed as a ‘sacred time’ to work on the self, whereby participants felt a mind–body connection. The four subthemes within this section are deeply interconnected, with the first component of this holistic wellbeing categorised as *improved psychological wellbeing*.

##### 3.2.2.1. Psychological wellbeing (subtheme 2.1)

The first dimension of this subtheme relates to personal growth. Participants experienced improved self-esteem and self-acceptance, whereby learning dance contributed to self-development and confidence (*“What this class has helped me achieve is, like, being more self-accepting,” Flora, FG2*). This self-development is also supported by changing attitudes to studying at university (*“taking that mentality into working on things at Uni was really good, really helpful,” Zara, Interview*).

Deeply intertwined with this was a sense that participating was a form of self-care, and applying this new act of self-care to wider life:

*“It was just also like making me remember I got to make time for myself I got to look after myself.” (Grace, FG2)*.

*“It’s nice to know that you can just do like a 10-minute exercise to get yourself into a positive mindset, and then get on with the rest of your day.” (Lily, Interview)*.

The second dimension of this subtheme explores how the classes were a distraction from having anxiety and from the stress of wider life pressures, helping to *“reduce stress”* and *“let go of worries” (Sophie, Creative reflection)* for a time. Participants did not feel that the classes improved their anxiety in every aspect of their lives necessarily (“*did not really do that much for me in terms of my anxiety*,” *Lily, Interview*), but that it gave them respite from it:

*“The dance classes have improved my wellbeing by reducing stress and making me feel calm. It has been a great opportunity to take myself away from my studies and ground myself.” (Sophie, Creative reflection)*.

*“The dance class offers a place where I can take my focus off my anxiety or worries and just concentrate on dancing and moving my body.” (Imani, FG3)*.

*“In terms of feeling anxious, it has been a tough time for me, but I have the hour and a half of dancing which I have to switch off from the world and actually focus on what I am doing (or I might fall over!!).” (Rosie, FG1)*.

One participant termed the dance class an “*outside source”* that supported them when in a *“dark anxious”* place, in contrast to the challenges of relying only on *“your own willpower” (Imani, Creative reflection)*. Others reported using resources from the class to support them with stress between classes (“*If I was just feeling stressed, I put on the [dance class] playlist*,” *Olivia, FG1*), thereby enacting the social identity of the group even when alone.

Distraction from daily worries and being immersed in the classes also improved mood. Participants used words such as *“chill” (Zara, Interview)*, *“calm” (Olivia, FG1; Bridget, FG3)*, *“feeling endorphins” (Cici, FG3)*, *“good energy” (Lily, Interview)*, *“optimistic” (Ana, FG1)* and *“positive” (Cici, FG3)*.

##### 3.2.2.2. Physical and bodily wellbeing (subtheme 2.2)

The classes also supported *improved physical and bodily wellbeing*. First, this was in relation to supporting the physical aspects of having anxiety:

*“… Maybe kind of the tension or like you know, just feeling all tense and the dance also, physically kind of you know loosened it up as we are moving… I felt kind of released everywhere where I hold usually the tension and also um like headaches…” (Ana, FG1)*.

*“I think people with anxiety often have physical symptoms of it in like hunching shoulders in belittling yourself. I know I pick at my nails I do lots of things. When you have to actually focus on what your body wants you know like my neck really wants some space right now and things like that.” (Mia, FG2)*.

Bodily tension was released through *“breathing and like stretching and release,”* with concentrating on dance *“slowing down the body and, a little bit, the heart” (Imani, FG3)*.

Second, the classes supported physical activity (“*gentle exercise,” Sophie, Creative reflection*), which helped participants prioritise exercise:

*“I really hate running and I’m not a jogging kind of person but it was like… my body was almost like craving physical movement in a sense that it hasn't before and so it's kind of just I feel like I’ve naturally just kind of developed an urge to move a little more.” (Flora, FG2)*.

*“I think it [dancing] has improved… physical health just because of flexibility Ian having to think about my body because I haven’t been exercising like I used to… paying attention to where my body is at and what It needs.” (Mia, FG2)*.

*“I think when life gets busy, I’m not so good at prioritising the things that look after my body, especially like exercise and movement. It just goes to the bottom of the pile. So, it’s good, it was good to have like, the weekly lessons because it reminds me that I do need to do something.” (Priya, FG2)*.

One participant said that doing exercises helped to *“clear my mind of stressful thoughts and directing my attention to the present moment” (Grace, Creative Reflection)*, suggesting a clear link between physical and psychological wellbeing. Another commented that doing physical exercise online changed their opinion of digital platforms, as they were more active than their usual Zoom use *(“aspects of growth for zoom and what’s possible,” Molly, FG2)*.

Intersecting closely with improved psychological wellbeing (subtheme 2.1), for some, the classes also improved body awareness and body confidence:

*“I’ve loved having the opportunity to regularly connect with my body and listen to it… I love having more awareness of my limbs, and how they fit into my personal space that surrounds me.” (Grace, FG2)*.

*“[The dance class] is allowing me to get out of my comfort zone, by experimenting with my body, getting me to feel and explore it in different ways than the ones I am used to. Getting to connect with my body in a new way, getting to challenge my body, is allowing me to cherish it more, to feel more comfortable in it.” (Molly, FG2)*.

*“… a way to remind myself that I live in a body, and that this body inhabits the world. And I have to pay attention to it sometimes.” (Mia, FG2)*.

Nonetheless, for one participant who had an underlying medical condition that made it harder for them to move their body in the way that they wanted to, they experienced challenges with feeling a sense of connection to their body:

*“I hated what it couldn't do. I thought dance would be a way for us [participant and their body] to learn to get on again, to support each other. For me to learn how to trust it again. But every week I'm freshly reminded of what I lost. I cry tears in between exercises when my body can't roll, or bend, or twist in the way I want it to.” (Maddie, Creative reflection)*.

##### 3.2.2.3. Social wellbeing (subtheme 2.3)

Closely interconnected with theme one and deeply intertwined with psychological and physical wellbeing, the next subtheme explores participants’ *social wellbeing*. Participants did not feel that the online classes created new friendships with other class participants, suggesting that an in-person class would have created stronger relationships. However, they felt an unconscious (*“without knowing it,” Flora, FG2*) sense of connection to others in the group:

*“…a fondness of other people rather than like a friendship.” (Bridget, FG3)*.

*“It has been very connecting to have a group of complete strangers who are all in the class for the same reason and nice to know that wherever they are in the world they are doing the same thing as me.” (Rosie, FG1)*.

*“And probably you know if it was in person, again, I would feel more connected to the others as well, but I felt some sort of, you know, connection.” (Ana, FG1)*.

Participants found it comforting to see *“a familiar face at the start” (Rosie, FG1)* of the classes, with one participant describing the group as *“like a little family” (Rosie, FG1)*, another feeling supported by the group (“*a group that kind of shielded me,” Molly, FG2*), and another stating that the classes supported with *“break[ing] away from [a] feeling of emptiness and isolation” (Flora, FG2)*. Moreover, many participants felt a connection with the dance practitioner who helped participants to feel energised and welcome:

*“You think of the people that you made a connection with – [dance practitioner], [dance moderator], umm people in the class – and, yeah, you think ‘I should go because these people are there’ and, I dunno, so, yeah, you do end up going and… you feel better afterwards.” (Mia, FG2)*.

*“I think like at the beginning of the process and straight off the bat, I did feel like connected to like [dance practitioner]. So say uhm she was once doing a lot and she herself is quite a welcoming person.”* (Imani, FG3).

Moreover, the classes seemed to support social confidence by focusing on the self in connection with others. As one participant described; *“the way our bodies can perform”* was seen to reduce the feeling of social anxiety through taking the focus away from comparison with others and onto *“the way I express myself” (Lily, Interview)*.

##### 3.2.2.4. Wider behavioural change (subtheme 2.4)

For some, the positive aspects of the classes on holistic wellbeing also impacted upon experiences outside of the dance classes, resulting in *wider behavioural change*. One participant described how learning from the dance practitioner made them feel more confident in other areas of their life:

*“I think, learning from [dance practitioner] has been very useful because she obviously knows herself and knows the way that her body works, well enough that she also know that whatever she’s doing, people aren’t looking at her differently because of it. She’s sort of owning what she does. I think I’ve learned a little bit of that. I’ve still got a like way to go but like, if I go and meet people in person I’m less conscious of how I’m moving and how I look outside. And so, and yeah, I think that’s something that has probably stuck with me from the process.” (Grace, FG2)*.

Other participants reported the influence of the classes on their student lives:

*“Remembering that just moving my body can be fun once a week, and that partly participating, or participating to the extent I can without becoming exhausted, is actually good, has been good for me, and helps me think positively about my social or academic participation.”* (*Kiera, Creative Reflection)*.

The classes also impacted routines, with one individual saying that “*it kind of gave me structure in other aspects of my life,”* such as with *“washing”* each week after class *(Grace, FG2)* and another noting that it gave them *“a reason” (Imani, Creative reflection*) to be more active. Several participants also described that they would like to take part in more artistic activities in the future, with one noting that they had already signed up for *“beginners’ salsa” (Grace, FG2)*.

##### 3.2.2.5. Structure and content of the online classes (subtheme 2.5)

Bringing all the subthemes together in this section, the last subtheme highlights how improvements in holistic wellbeing were underpinned by the *structure and content of the online classes*. First, this was in relation to the weekly structure of the classes that provided consistent support for participants. The classes provided *“a form of structure”* in among the *“chaos”* of *“personal life” (Rosie, FG1)* and added *“more structure”* to one evening a week *(Imani, FG3)*.

Second, the content of the classes including shared drawing exercises and learning dance skills, were viewed as key components that contributed to wellbeing:

*“I also like the drawing at the beginning of the class because I never have time to draw and I loved it as a kid.” (Ana, Creative reflection)*.

*“I was happy because I was getting better at remembering the choreographies or like the exercises.” (Ana, FG1)*.

Many participants also agreed that it was the focus of the classes on inclusivity and a movement away from perfection that facilitated enjoyment, whereby it did not matter how ‘good’ one was at dancing. However, there were mixed views about having breakout rooms to talk to other participants (*“just found it hard,” Ana, FG1*; *“nice to have the breakout rooms,” Zara, Interview*), with some feeling that you cannot replace in-person socialising (*“in-person would have helped me more,” Molly, FG2*). Similarly, there were mixed feelings in relation to the online format of the classes, with some feeling that being online was secondary to an in-person class and others reaping benefits from being in their personal spaces and finding it more accessible (*“more effective if it was in person but… Zoom allows you to be in a place where you are already comfortable,” Molly, FG2*).

### 3.3. Quan findings

#### 3.3.1. Descriptive statistics

Quantitative data were available for 59 data points: 27 participants completed week 1 surveys, 18 participants completed week 4, and 14 participants completed week 8. The descriptives across the three timepoints, presented as means and standard deviations (SD), are shown in Table 4.

**Table 4:** Descriptives of quantitative measures across the eight weeks.

	Week 1 (*n* = 27)	Week 4 (*n* = 18)	Week 8 (*n* = 14)
	**Mean (SD)**		
**Anxiety**	11.33 (5.26)	9.11 (4.20)	7.43 (2.74)
**Loneliness**	6.37 (1.62)	5.33 (1.71)	5.0 (1.71)
**Depression**	2.52 (1.78)	1.94 (1.70)	1.43 (1.16)
**Wellbeing**	21.26 (4.03)	21.67 (3.74)	22.93 (2.67)
**Self-esteem**	3.67 (1.92)	3.83 (1.54)	4.21 (1.19)
**Self-efficacy**	15.44 (3.24)	16.0 (2.43)	17.21 (2.78)
**Trust in teacher**	5.78 (1.40)	6.33 (0.84)	6.50 (0.94)
**Group closeness**	2.67 (1.39)	4.06 (1.35)	4.71 (1.49)
**Group support**		5.17 (1.50)	5.36 (1.60)
**Group identity**		4.82 (0.95)	5.14 (1.45)
**Collective efficacy**		5.61 (0.98)	5.57 (1.40)

SD = standard deviation.

#### 3.3.2. Changes in mental health, wellbeing, and loneliness

RM ANOVA results are presented in-text. See Table 5 for pairwise comparisons of predictive margins indicating when changes occurred across the 8 weeks and in which direction. Sensitivity analyses mirrored these findings apart from wellbeing (please refer to Supplementary Table S2).

**Table 5:** Margins and pairwise comparisons for week 1, week 4, and week 8 (*n* = 14).

	Week 1			Week 4	Week 8	Week 1 vs. Week 4			Week 1 vs. Week 8			Week 4 vs. Week 8		
	Margins (predicted means)					Contrasts	95%CI	*p* - value	Contrasts	95%CI	*p* - value	Contrasts	95%CI	*p* - value
**Anxiety**	11.14			8.43	7.43	**−2.71**	**−4.91, −0.51**	**0.018**	**−3.71**	**−5.91, −1.51**	**0.002**	−1.00	−3.20, 1.20	0.359
**Loneliness**	6.14			5.07	5.00	**−1.07**	**−1.85, −0.3**	**0.009**	**−1.14**	**−1.92, −0.37**	**0.005**	−0.07	−0.85, 0.70	0.851
**Depression**	2.43			1.86	1.43	−0.57	−1.29, 0.15	0.115	**−1.00**	**−1.72, −0.28**	**0.008**	−0.43	−1.15, 0.29	0.232
**Wellbeing**	21.07			22.14	22.93	1.07	−0.62, 2.77	0.205	**1.86**	**0.16, 3.55**	**0.033**	0.79	−0.91, 2.48	0.349
**Self-esteem**	3.36			3.71	4.21	0.36	−0.24, 0.96	0.232	**0.86**	**0.26, 1.46**	**0.007**	0.50	−0.10, 1.10	0.098
**Self-efficacy**	15.07			15.93	17.21	0.86	−0.22, 1.94	0.114	**2.14**	**1.06, 3.22**	**<.001**	**1.29**	**0.21, 2.36**	**0.021**
**Trust in teacher**	6.07			6.50	6.50	0.43	−0.08, 0.93	0.092	0.43	−0.08, 0.93	0.092	0.00	−0.50, 0.50	1.000
**Group closeness**	2.86			4.36	4.71	**1.50**	**0.64, 2.36**	**0.001**	**1.86**	**0.99, 2.72**	**<.001**	0.36	−0.51, 1.22	0.403
**Group support**				5.50	5.36							−0.14	−0.89, 0.60	0.686
**Group identity**				5.11	5.14							0.04	−0.59, 0.67	0.904
**Collective efficacy**				5.57	5.57							0.00	−0.45, 0.45	1.000

Predicted margins (means) of each measure at each timepoint are presented in the left-hand side of the table for participants with complete cases (*n* = 14). Pairwise comparisons of these margins are presented in the remainder of this table where contrasts (differences) between margins across weeks are presented, alongside 95%CI (95% confidence intervals) and *p*-values for *n* = 14. Estimates in bold indicate where there is evidence of change between weeks. Margins, contrasts, and 95%CI are rounded to 2 decimal places.

For *anxiety*, there was evidence of differences across the eight weeks, *F*(2, 26) = 6.45, *p* = .005. Pairwise comparisons suggest that anxiety reduced between week 1 and week 4, week 1 and week 8, but not between week 4 and week 8. For *loneliness*, there was evidence of differences across the eight weeks, *F*(2, 26) = 5.77, *p* = .008, suggesting a reduction between week 1 and week 4, week 1 and week 8, but not between week 4 and week 8. For *depression*, there was evidence of differences across the eight weeks, *F*(2, 26) = 4.11, *p* = .03, with a reduction between week 1 and week 8 suggested, but not between week 1 and week 4 or week 4 and week 8.

For *wellbeing*, there was evidence of differences across the eight weeks, *F*(2, 26) = 2.56, *p* = .09, with an increase between week 1 and week 4 only. For *self-esteem*, there was evidence of differences across the eight weeks, *F*(2, 26) = 4.36, *p* = .02, with an increase between week 1 and week 8 only. For *self-efficacy*, there was evidence of differences across the eight weeks, *F*(2, 26) = 8.45, *p* = .001, indicating an increase between week 1 and week 8 and week 4 and week 8, but not between week 1 and week 4.

#### 3.3.3. Changes in group processes

For *trust in the teacher*, there was no evidence of difference over the eight weeks, *F*(2, 26) = 2.03, *p* = .15. For *group closeness*, there was evidence of differences across the eight weeks, *F*(2, 26) = 11.00, *p* < 0.01, indicating an increase between week 1 and week 4, week 1 and week 8, but not between week 4 and week 8. There was also no evidence of difference across week 4 and week 8 for *group support F*(1, 13) = 0.17, *p* = .685, *group identity F*(1, 13) = 0.01, *p* = .904, or collective efficacy *F*(1, 13) = 0.00, *p* = 1.00. Ceiling effects were evidenced (i.e., >20% of participants scoring the highest) for trust in the teacher, group support and collective efficacy across all timepoints. This helps explain why evidence of change was not shown.

## 4. Discussion

This study applied the theoretical lens of the social cure to the context of online dance classes for young people living with anxiety, exploring if and how online classes support mental health and loneliness through the co-construction of shared group identity and associated psychological resources (e.g., collective efficacy and group support). Through our qualitative analyses, we found that participants did construct a shared identity, but this was grounded in shared experience rather than feelings of ‘membership’, which is often attributed to strong group identities within social identity theory. This was, in part, supported by our quantitative data. While there was no evidence of statistical change to group identity, group identity was already very high at week 4. Our qualitative analyses also revealed that participants experienced improvements in their holistic wellbeing, providing a sense of positive mind–body connection, also complemented by increases in wellbeing seen in our quantitative data. In turn, reductions in anxiety, depression, and loneliness and increases in self-esteem, self-efficacy, and group closeness were found in statistical analyses. The quantitative findings largely converged with the qualitative findings but with some nuances around anxiety and depression (e.g., there was a less direct articulation of this in focus groups).

### 4.1. Expanding the social cure

This is one of the first known studies to assess whether online group dance can support young people’s mental health and loneliness. Additionally, it is one of only a handful of studies that have applied the social cure framework to arts and health. Certain dimensions of our research align with these previous studies. For example, several previous studies highlight the role of arts-based groups in eliciting social identity with their groups (Williams et al., 2019; Dingle et al., 2020; Forbes, 2021), agency (Forbes, 2021), belonging, support, and purpose (Williams et al., 2020; Forbes, 2021), positive emotions and self-efficacy (Williams et al., 2020), and improved wellbeing (Williams et al., 2019). However, this study builds on these findings and stands to make a unique contribution.

In qualitative findings, rather than a focus on ‘internalised group membership’ as a mechanism for improved mental health, we found that some participants redefined membership as ‘team’ and ‘nurturing environment’ and rejected the language of ‘belonging to the group’. One reason for this could be that our study took place online. This suggests that the absence of physical co-presence among participants may minimise the ‘risk’ of creating strong exclusionary boundaries (as is theorised in social identity theory in relation to how ‘out groups’ are created) (Jetten et al., 2012; Haslam et al., 2018). To participants, the online ‘environment’ had fewer boundaries than a ‘group’, keeping the class open for all members to come together in diversity rather than sameness. Participants also felt that the online space allowed them to choose how they wanted to engage, such as whether to have their cameras on. Other factors may have also contributed to a sense of inclusivity, such as the dance practitioner’s approach and dance style. Previous research has highlighted the challenges of strong membership groups that may exclude those who do not identify (e.g., creating “barriers to outsiders”) (Collins, 2004), and our research suggests that online engagement may minimise this risk when delivered as part of carefully designed and facilitated beginner classes. Although belonging was not explicitly measured in surveys, there was descriptive evidence of group processes (i.e., group identification, collective efficacy) being present in week 4. So, one possible explanation is that group mechanisms were at play and that it was simply the language used by the participants that did not align with the theoretical literature rather than the participants’ experiences themselves. Exploring this further, complexity exists beneath the surface of perceptions of this inclusive environment. First, one participant did feel excluded due to the lack of ethnic diversity in their class. Second, other affiliations (such as institutions) played a role in the positive experience of the classes, and not everyone had already built these previous connections.

Further, participants not identifying with the language of ‘belonging’ may be due to a weaker sense of membership online when compared to face-to-face engagement. Several participants reported that meeting in person would have increased opportunities for friendships rather than ‘familiarity’, as was created through the classes. It was highlighted that instead of ‘belonging’ as a key mechanism, it may have been ‘group closeness’. Our quantitative findings supported this, and qualitatively, participants reported feeling closer to others in the group and to the organisations of UCL and Dance Base, even if they did not feel something as strong as ‘belonging’. This is supported by Draper and Dingle (2021), who found that group identification and psychological needs satisfaction were lower for groups in virtual modes versus in-person modes, explored in response to pre-existing dance, music, and instrumental groups going online due to COVID-19 (Draper and Dingle, 2021). While the online groups did provide group and individual benefits aligning with the social cure, they were weaker than face-to-face engagements (Draper and Dingle, 2021). This may be what we are seeing here and why participants do not align with the language of ‘belonging’ but with group closeness.

Nonetheless, our study further unpacks the nuances of online engagement, highlighting the specific benefits of online engagement over in-person. For example, the shared online environment enabled participants to focus more on their own experience and their bodies, providing a strong sense of mind–body connection. Online engagements may be more accessible, such as for those with social anxiety or when other factors affect in-person engagement (e.g., location), as meeting online provides control over the environment that is not possible in person and can be more easily accessed. This is reflected in our sample, where approximately 30% of participants who joined the first dance class engaged with arts activities a few times a year or less before the outbreak of COVID-19. Our research expands the social cure by suggesting that, for some, this control of the environment is an important aspect of identifying with the ‘team’ because it deepens a sense of inclusivity in relation to who can access the class and benefit from it.

Another development was in relation to the role of the dance practitioner. Qualitative analyses showed that they played a key role in encouraging and energising participants and, while our quantitative measure of trust in teacher did not show evidence of statistical change, descriptively it was high after the first class and maintained across the 8 weeks. Tarrant et al. (2020) have previously explored how group facilitators can consciously support the development of meaningful social identities (Tarrant et al., 2020). This has included recognising the importance of supporting interaction between group members and encouraging cooperation, trust, interest, involvement, and social connectedness (Tarrant et al., 2020). Given that we worked with our dance facilitator to develop a class structure that promoted social connections (e.g., including time for break-out room discussions), our study can be seen as aligning with this broader research that suggests there may be specific facilitation styles that can encourage identity-building. Sociological research can also support us in understanding the importance of this finding. Weber (1968) put forward the notion of ‘charismatic authority’ (a kind of leadership founded upon charisma), which has been built on in research examining the mechanisms of strong group solidarity (Weber, 1968). Notably, Collins and McConnell (2015), Collins (2019, 2020) has suggested that individuals with charisma set the emotional tone of interactions, supporting participants in a group to feel ‘pumped up’ with energy (“filling them with enthusiasm” 2019, p. 48), with this energy playing a key part in creating group identification (Collins and McConnell, 2015; Collins, 2019, 2020). Connecting this to social identity theory, it suggests that energising participants in this way, where they feel a kind of ‘admiration’ for the practitioner that motivates and energises them, may contribute to a shared group identity that links in with the acquisition of psychological resources.

Another key reflection is linked to our finding of wider behavioural change in relation to psychological resources. We found that participants acquired several psychological resources identified within social cure theorising, such as a sense of meaning, motivation, social support, and personal agency. Notably, we found evidence of statistical increases in our measures of self-efficacy and self-esteem. We also learned more about how these new resources played a role in the broader lives of our participants, thereby supporting their overall health. Wider behavioural change has been seen in other studies exploring arts interventions for mental wellbeing (Warran et al., 2019). However, to the authors’ knowledge, this has not been connected to the theorising of the social cure. Thus, our study shows that there may be important behavioural mechanisms (e.g., changes to social and academic behaviours that develop outside the dance classes, which underpins improved holistic wellbeing.

### 4.2. Divergent findings

There are also several interesting points of departure between our qualitative and quantitative findings. First, anxiety was shown to reduce statistically, but in focus groups, participants were cautious of saying they felt their anxiety had been reduced. They did feel that the classes helped with anxiety symptoms (e.g., physical tension), but their focus was more on respite from anxiety rather than minimising it. We may see these slightly different results because participants may have felt in-the-moment relief from their anxiety immediately after the classes (reflected in lower anxiety scores) but were unable to contextualise this in their wider lives, where, in the focus groups, participants acknowledged that relief from anxiety may not have the longevity of changing longer-term anxiety. Another explanation may be that participants were speaking of ‘anxiety’ in relation to different feelings than measured in the GAD-7, which does not explicitly ask about somatic symptoms of anxiety (e.g., tension, breathing) or social anxiety. In contrast, these themes were present in the qualitative discussions.

Second, we found that depression was reduced statistically, but participants did not talk about depression in the focus groups/interviews. As anxiety and depression are often co-morbid (Cummings et al., 2014), it seems unusual for there to be no subjective connections made by our participants. It remains for future research to explore whether the statistical reductions we have found in our dance classes may have the potential to build up over time to lead to a longer-term subjective positive impact on those living with anxiety, as well as exploring further this complex relationship between anxiety and depression in the context of online dance for this population.

### 4.3. Strengths and limitations

There are many strengths to our study. Notably, this is the first study that has (i) applied the social cure approach in the context of delivering online dance classes and (ii) explored the role of online dance in young people’s mental health. It is also methodologically innovative, combining participatory, qualitative, and quantitative methodologies in a triangulation design framed within the theoretical framework of the social cure. Further, it was a responsive study to the rapidly changing social conditions of the COVID-19 pandemic, providing immediate support for young people during a time when mental health challenges were rising. This unique study has provided novel findings and theoretical developments that can practically support developing online arts-based interventions in the future and deepen our understanding of how the social cure operates in an online context. It has contributed to our understanding of the specific benefits of dance – an under-researched area in young people’s mental health, which we have shown has unique benefits, such as improving body acceptance and learning dance skills.

However, the study also has several limitations. First, our sample demographics are not representative of the UK population as our sample was predominantly white, identified as female and indicated high socioeconomic status. Given that one of our participants from an ethnic minority group had feelings of exclusion from some aspects of the dance classes, it is vital that further research in this area acknowledges the barriers and enablers of online dance participation and connected research, as has been a priority area in recent years (Mak et al., 2020). This should include reflecting on intersectional identities across age, gender identity, sexual orientation, socioeconomic experiences, race, culture, religion, and other categories of ‘difference’ to disrupt power dichotomies and strengthen the equity of access to this potential mental health support for all young people. This should also extend to establishing diverse research teams and facilitators, ensuring that diversity is considered in every stage of project development and delivery and that researchers engage in critical reflection of their positionality and lived experiences. Second, due to recruitment challenges and the short-term nature of this project, we operated primarily on a convenience sampling basis, which meant that most of our participants were affiliated in some way with the organisations delivering this project. While this may have had positive implications regarding the sense of identification and trust that participants had in the project, it limits our understanding of whether we would have found the same positive benefits across a more diverse sample. Third, the design of the embedded quantitative component has methodological limitations. Despite the sample size being appropriate to engage in theoretical innovation and to explore subjective lived experiences qualitatively, it is small from a quantitative perspective. Further, a control group comparison is needed to explore whether statistical changes across measures are being seen (rather than regression to the mean). Randomisation would also help to protect against selection bias to ensure an equal likelihood of different people engaging in the study (e.g., rather than only people who already engage in arts activities or are healthier taking part). Lastly, surveys were measured in week 1 after the first dance class, so we are unable to look at measurements before the dance classes began.

This study has highlighted the potential benefits of online dance classes for young people living with anxiety. Therefore, one avenue for future research would be to conduct a randomised controlled trial in a larger and more representative sample that omits some of the mentioned biases. Within this, there could be a comparison of online to in-person engagements. Doing so could help to further understand how to optimise similar arts-based activities and interventions for young people, such as for Social Prescribing schemes. Another avenue could be to explore the application of the social cure to a broader range of online arts-based interventions to see whether the theoretical developments we have constructed through this study would be relevant in the context of other kinds of engagements. Finally, we do not know from our study exactly when (e.g., after how many weeks of engagement) changes to mental health, wellbeing, loneliness, and group processes started to occur. It remains for future research to explore longer-term dance interventions to understand how to best ‘match’ the length of online classes to those experiencing anxiety symptoms.

### 4.4. Conclusion

Overall, this study theoretically expands the social cure for young people living with anxiety in the context of online dance by redefining ‘group membership’ (in view of the language of ‘team’ and ‘environment’), unpacking the potential benefits of online interventions (e.g., in relation to accessibility, control of the environment, and facilitating a mind–body connection), spotlighting the vital role of the dance practitioner in setting the emotional tone of group interactions; and highlighting how behavioural changes (e.g., social and academic behaviours) are important for improved social and mental wellbeing. It also demonstrates the potential for online group dance to reduce anxiety, depression, and loneliness, improve wellbeing, self-esteem, and self-efficacy and foster group closeness. Nonetheless, research needs to ensure that online dance interventions are more accessible, diverse, and inclusive.

## Data availability statement

The datasets presented in this article are not readily available because the raw data from this study is not being publicly archived for use by other researchers because the data contains information that could compromise the privacy of research participants. The UCL Research Ethics Committee have restricted the use of the data to UCL researchers only. To discuss the conditions of this availability further, please email the authors. Requests to access the datasets should be directed to k.warran@ucl.ac.uk.

## Ethics statement

The studies involving humans were approved by the University College London (UCL) Research Ethics Committee (Project ID: 19105/002). The studies were conducted in accordance with the local legislation and institutional requirements. The participants provided their written informed consent to participate in this study. Written informed consent was obtained from the individual(s) for the publication of any potentially identifiable images or data included in this article.

## Author contributions

SF: Conceptualization, Data curation, Formal analysis, Funding acquisition, Investigation, Methodology, Project administration, Writing – original draft. LW: Conceptualization, Data curation, Formal analysis, Funding acquisition, Investigation, Methodology, Project administration, Writing – review & editing. HM: Conceptualization, Data curation, Formal analysis, Funding acquisition, Investigation, Methodology, Project administration, Writing – review & editing. EÅ: Project administration, Writing – review & editing. LN: Formal analysis, Writing – review & editing. GD: Methodology, Writing – review & editing. KW: Conceptualization, Data curation, Formal analysis, Funding acquisition, Investigation, Methodology, Project administration, Supervision, Writing – original draft.
