# Supplementary material for: Expanding the social cure: a mixed-methods approach exploring the role of online group dance as support for young people (aged 16–24) living with anxiety
Source: Front Psychol. 2023 Oct 17;14:1258967. doi: 10.3389/fpsyg.2023.1258967 (PMC10616254; doi:10.3389/fpsyg.2023.1258967)
Supplement: Supplementary file 1 [file Data_Sheet_1.PDF]

Supplementary

Recruitment process

Recruitment involved the following activities: phone calls, emails, and e-flyers to schools, colleges, and charitable organisations who work with young people or those requiring mental health support; social media activity, particularly targeted at mental health, arts and health, and youth networks; inclusion in relevant e-newsletters (e.g., from the UKRI MARCH, Loneliness and Social Isolation in Mental Health, and TRIUMPH networks); and contact with student unions, including presence at university freshers' weeks (notably UCL, University of Edinburgh and the University of Glasgow).

Structure of online dance classes

Table 1. The approximate structure of the dance classes across 90 minutes.

Timings	Structure	Content
0-5 mins	Introductions and start of classes	Participants entered the Zoom room where a light-hearted song was played as the 'entrance song'. This was turned down when everyone joined. The moderator took a class register. In the first couple of classes, the dance practitioner and moderator introduced themselves, thereafter classes started with a conversation between them once people had entered the room.
5-15 mins	Ice-breaker drawing tasks	Participants took part in an icebreaker drawing task with a different theme each week (e.g., drawing something they like). Participants, the dance practitioner, and the moderator would hold up their drawings to the screens and the dance practitioner would share some thoughts on the drawings. Sometimes participants would comment, such as guessing what drawings were supposed to be or describing what the drawing represented for themselves.
15-20 mins	Improvisation and imagery tasks	The dance practitioner would share an inspiration/theme for the class. This was usually imagery that participants could think about when dancing through the class (e.g., imagine your head is in a spacesuit, your bones are fluid like liquid etc.). Following this, a YouTube video and track of music were played for 3-5 mins and everyone was invited to move freely, thinking about that imagery. This responsive, improvisation-based task builds on somatic and contemporary techniques that lead with the physical body and feelings rather than what something looks like or the technique that should be followed. The dance practitioner deliberately chose to start classes this way to encourage the participants to let go of the inhibitions of moving.
20-75 mins	Dance and movement exercises followed by creative tasks	The dance practitioner led a contemporary-dance style class, with adaptations for working online. This started with a set of warm-up exercises that involved spine mobilisation (spine 'roll down') and some stretches. This was followed by the main dance movements, which included leg bends in different positions (pliés), arm mobilisation and arm swings, foot exercises including leg stretches and balancing on tip-toes, small jumping exercises, transfer of weight exercise, and a gestural hand phrase that became a base for a creative task (see below). These movements were repeated over the course of the 8-week block, sometimes with developments and music changes to test out different dynamics. The exercises represent a typical structure for a contemporary dance class and incorporated a lot of techniques and approaches found in contemporary-style dance classes (with influences from Ballet, Cunningham, Limon, and somatic/release techniques, amongst others). The dance practitioner used an eclectic mix of music, including pop, country, and instrumental – and closer to the winter holiday used festive tunes at the request of participants.<br>At the end of the class, the dance practitioner led a creative task with the participants, which was developed with the classes and responded to the participant's needs. Some examples of creative exercises included: participants created additional gestures to add to the dance practitioners gesture phrase, which were shared with each other; participants were invited to move their camera to show only their feet – a short 'foot choreography' was created and some participants wore shoes for this task to add character; and participants worked together in small breakout room groups to create something collectively in response to the task.
75-90 mins	Chats, watching dance films and discussions	After the above, there was some space for participants to chat. It was sometimes felt to be appropriate for breakout rooms where smaller groups could chat, sometimes the group talked as a whole. The dance practitioner invited discussions about some of the clips that had been watched or about a particular exercise, asking participants to feedback to the group on how they felt or what they were thinking. In the last few minutes, any housekeeping or any updates from the researchers were shared.

Demographic and health measures

Gender
Participants were asked ‘Which gender do you most identify with?’ and were provide with single answer options of ‘Female’, ‘Gender non-conforming’, ‘Male’, ‘Non-binary’, ‘Transgender’, or ‘Prefer not to say’. There was also an open text box after the item ‘If you prefer to use your own term, please specify in the box below’.

Ethnicity
Participants were asked ‘Which ethnicity group do you most identify with?’ and were provide with single answer options of ‘Welsh/English/Scottish/Northern Irish/British’ , ‘Irish’, ‘Gypsy or Irish Traveller’, ‘Any other White background’, ‘White and Black Caribbean’, ‘White and Black African’, ‘White and Asian’, ‘Any other Mixed/Multiple ethnic background’, ‘Indian’, ‘Pakistani’, ‘Bangladeshi’, ‘Chinese’, ‘Any other Asian background’, ‘African’, ‘Caribbean’, ‘Any other Black/African/Caribbean background’, ‘Arab’ or ‘Prefer not to say’. There was also an open text box after the item ‘Any other ethnic group, please describe below if you wish’.

Parental/carer education
Participations were asked about their parental education to assess their socioeconomic position. This was because education and income across the age range of 16-24 years would vary substantially. Participants were asked ‘This question asks you about the person or people who are responsible for your care (or have been in the past), such as a carer/guardian/parent. You can choose to answer one question or two depending on your circumstances (if this is not applicable, please select the third column).’. The question was repeated twice for parent/carer 1 and parent/carer 2. Answer options included ‘School and college qualifications (for example GCESs, A-Levels or equivalent)’, ‘Undergraduate degree (or equivalent) or above’ or ‘I do not know, prefer not to say or not applicable’.

Lived experience of physical health, mental health, and disability
Participants were asked ‘Do you have a physical health condition (e.g., asthma, diabetes, migraines, skin disorders)? “, ‘Do you have a mental health condition (e.g., depression, anxiety)?’ and ‘Do you have any disabilities?”, for physical health, mental health, and disability, respectively. Answer options included ‘No’, ‘Yes’, and ‘Prefer not to say’.

Arts behaviours
Frequency of engagement in arts activities was asked. This included general arts engagement before the COVID-19 pandemic: ‘Before the COVID-19 pandemic began in March 2020, how often would you say you engaged in these arts/creative activities generally?”. Answer options included ‘Never or rarely’, ‘Less than once a year’, ‘A few times a year’, ‘At least once a month’ or ‘At least once a week’.

Participants were also asked any changes to arts behaviours throughout the pandemic: ‘Thinking about your engagement levels throughout the COVID-19 pandemic (i.e., since March 2020), has there been any change in your levels of engagement with arts/creative activities?’. Answer options included ‘I engage less than before the pandemic’, ‘There has been no change’, ‘I engage more than before the pandemic’.Participants were also asked about changes to online arts behaviours since the pandemic: ‘Since the COVID-19 pandemic started in March 2020, how has your online engagement with these arts/creative activities changed?’. Answer options included: ‘I engage less’, ‘There has been no change’, or ‘I engage more’.

Semi-structured interview guide

1. **We invite everyone to introduce themselves and to reflect on one thing you have done outside of the dance classes in the last 8 weeks that supported you to feel creative and well** [Go around everyone in the group. Allow people to type or opt out of introducing themselves if they don't feel comfortable]

River journey exercise

- Ask participants to close their eyes and envision a beautiful river, flowing from the start to the present and into the future.
- Ask participants to reflect on how they were feeling when they first joined the dance classes, in the middle, and what they feel now, coming to the end of the 8 weeks. What are some of the most significant experiences and/or changes in relation to your wellbeing or your social relationships with others in the group?
- Invite participants to take out a piece of paper and coloured pens/pencils/pastels if they have them. Ask participants to draw a long windy river across their page. Ask them to write start on the left side, middle in the centre, and present on the right [at this point we will share an example of what their river might look like], and then to spend five to fifteen minutes to draw or write out their reflections/experiences along the river line.

Semi-structured questions

General questions

2. **Would anyone like to share their reflections on drawing out their river?**
   Prompts: What came to mind? What did you write/draw and why? Would anyone like to hold up their picture and describe it?
3. **How would you describe your experience of engaging with the online dance classes?**
   Prompts: What words come to mind? Why?

Group membership

4. **How did you find engaging in an online group setting?**
   Prompt: did you focus mainly on the dance practitioner or did you look at others? Did you use your camera or not, and why – did it make a difference to you? What made you feel present in the moment and focused during the classes? What distracted you?
5. **How would you describe the dance class - would you say that it's a group, class or community?**
   Prompts: What word would you use? Why?
6. **Do you feel that you belong to the dance group? Or would you use another word other than belong?**
   Prompt: If yes, what aspects of the class have supported you to belong - the dance practitioner, other members of the class, the choice of dancing? If no, why not – what prevented you from feeling that you belong to the group?
7. **How did you find using the online platform Zoom to connect for the classes?**
   Prompts: Did it help or hinder you to feel connected to the group? What worked and what didn't?
8. **What do you think of what you hear the words Dance Base / UCL / Dance/Connect?**
   Prompts: Do you feel connected to these words or not? Do you feel a sense of belonging when you hear them or not, and why?

9. **When you think about what defines who you are, would you include dancing or being a member of these dance classes in your list?**
   Prompts: Do you identify with the group? Why? Why not?

Social wellbeing

10. **Would you say that you feel a sense of connection to others in the group or not?**
    Prompts: Why? What supported or lessened these feelings of connection? Have the classes supported you to feel less lonely or isolated?
11. **Would you say that you have formed any new relationships such as friendships through engaging with the classes?**
    Prompts: If yes, what supported the formation of these relationships? If no, what prevented them?
12. **Has anyone communicated with one another outside of the dance class?**
    Prompts: How have you connected? Will you stay in touch with one another?

Mental wellbeing

13. **How did attending the online dance classes make you feel? [note that these could be positive or negative and remind participants this is an open space for dialogue]**
    Prompts: What emotions have you experienced across the 8 weeks? Have they supported you to feel confident / happy / sad / excited / tired / energised? What aspects of the classes prompted these feelings do you think?
14. **Research shows that dance classes may be able to support young people living with anxiety. What do you think about this? Is this your experience or not?**
    Prompts: did engaging with the classes make you feel more relaxed? Did they reduce any of your normal symptoms of anxiety such as feeling irritable or feelings of panic?
15. **If the classes did support you in relation to your anxiety, why do you think this was the case?**
    Prompts: what was it specifically about the classes that helped? The structure? The people? The choreography? The online platform? The music? Belonging to a group? Being part of something?

Social support

16. **Have the dance classes supported you in any way? If yes, how?**
    Prompts: do you feel supported by others in the group? Also note examples such as providing support during uncertain times, finding connection during times of isolation etc.

Barriers

17. **Have you experienced any barriers to engaging fully in the classes?**
    Prompts: For example, was there anything about the class you found more difficult to engage with, or was there anything that may have prevented you from attending long-term? Anything about the class structure or content that may be a barrier?

Research participation

18. **How did you find the experience of engaging with the research?**
    Prompts: experiences of completing the surveys/ journal entries/ writing for the blog

Close

19. **Do you have any other comments? Is there anything else you feel is important for us to know to support this research study?**

20. Do you have any questions for us?

Ask participants to take a picture of their rivers and send them to us via UCL Dropbox (if they feel comfortable doing so). Thank participants for their time.

Ethnographic reflections template

**Date of class**	
Number of attendees	
Any notes about logistics/delivery/tech issues	
Were people in the class focused and engaged or did you notice anyone wandering out of their camera or not really paying attention? Or were most cameras off?	
What was the general mood/feel of the class?	
What, if anything, did you notice about young people's confidence in contributing to the creative and physical exercises in the class?	
If people engaged in any conversation, what kinds of things did they talk about? Have these conversations changed throughout the course?	
Did you observe anything else that may be relevant to our understanding of whether the dance classes: 1) improve mental and social wellbeing, and 2) help to construct a sense of belonging to the dance group?	
Any other comments	
Completed by	

Coding framework for qualitative transcripts

**GROUP**
Group membership
Group belonging
Social identity
Social support
Connection
Connection to dance practitioner
**MEANING**
Meaningful relations
Meaningful experiences
Collective meaning
**INDIVIDUAL**
Self-identity
Sense of purpose
Personal agency/control
Motivation to help others
Confidence
**MENTAL HEALTH**
Self-efficacy
Reduced loneliness
Improved mood
Improved wellbeing
Reduced depression

Quan measures

Anxiety

Questions asked, “Over the last 2 weeks, how often have you been bothered by any of the following problems?” (Spitzer et al., 2006). Items included: i) “Feeling nervous, anxious or on edge”, ii) “Not being able to stop or control worrying”, iii) “Worrying too much about different things”, iv) “Trouble relaxing”, v) “Being so restless that it is hard to sit still”, vi) “Becoming easily annoyed or irritable”, vii) “Feeling afraid as if something awful might happen”. Response options included and were scored as the following: “0=Not at all”, “1= Several days”, “2=More than half the days”, and “3=Nearly every day”. Scores were summed to create an overall anxiety score, ranging from 0-21, with higher scores indicating higher anxiety. Cut-off points to indicate different levels of anxiety include <5 for no anxiety, 5-10 for mild anxiety, 10-15 for moderate anxiety and >15 for severe anxiety (Spitzer et al., 2006).

Loneliness

Questions asked, “Please say how you have been feeling in the past 2 weeks about each sentence”, which included: i) “How often do you feel that you lack companionship?”, ii) “How often do you feel left out?”, iii) “How often do you feel isolated from others?” (Hughes et al., 2004). Response options included and were scored as the following: “1=Hardly ever”, “2=Some of the time”, and “3=Often”. Scores were summed to create an overall loneliness score, ranging from 3-9, with higher scores indicating higher loneliness (Hughes et al., 2004).

Depression

Questions asked, “Over the last 2 weeks, how often have you been bothered by any of the following problems?” (Kroenke, Spitzer & Williams, 2003). Items included: i) “Little interest or pleasure in doing things” and ii) “Feeling down, depressed, or hopeless”. Response options included and were scored as the following: “0=Not at all”, “1= Several days”, “2=More than half the days”, and “3=Nearly every day”. Scores were summed to create an overall depression score, ranging from 0-6, with higher scores indicating higher depression (Kroenke, Spitzer & Williams, 2003).

Wellbeing

Questions stated “Below are some statements about feelings and thoughts. Please say which best describes your experience of each over the last 2 weeks” (Anon, 2008). The seven Items included: i) “I’ve been feeling optimistic about the future”, ii) “I’ve been feeling useful”, iii) “I’ve been feeling relaxed”, iv) “I’ve been dealing with problems well”, v) “I’ve been thinking clearly”, vi) “I’ve been feeling close to other people”, vii) “I’ve been able to make up my own mind about things”. Response options included and were coded as the following: “1=None of the time”, “2=Rarely”, “3=Some of the time”, “4=Often”, “5=All of the time”. Scores were summed to create an overall wellbeing score, ranging from 7-35, with higher scores indicating higher wellbeing (Anon, 2008).

Self-efficacy

Questions asked, “Please indicate which best describes your opinions” in regard to six items: i) “If someone opposes me, I can find means and ways to get what I want”, ii) “I am certain that I can accomplish my goals”, iii) “I am confident that I could deal efficiently with unexpected events”, iv) “Thanks to my resourcefulness, I know how to handle unforeseen situations”, v) “I can remain calm when facing difficulties because I can rely on my coping abilities”, vi) “No matter what comes my way, I’m usually able to handle it” (Romppel et al., 2013). Response options included and were coded as the following: “1=Not at all true”,

"2=Hardly true", "3=Moderately true", and "4=Exactly true". Scores were summed to create an overall self-efficacy score, ranging from 6-24, with higher scores indicating higher self-efficacy (Romppel et al., 2013).

Trust in teacher

This was measured by creating a one-item question: "This question asks you to rate your trust in the dance class teacher - I have trust in the teacher". Participants rated this on a seven-point Likert scale, ranging from "1=Not at all" to "7=Very much". With higher rating indicating higher trust in teacher.

Group closeness

This was measured using the Inclusion of Other in Self Scale (IoS scale), which is a graphical representative of closeness (Aron, Aron & Smollan, 1992). This was adapted to ask "Looking at the image of the circles above, which picture best describes your relationship with the dance group? (Self here refers to you as a member of the dance class.)". Circles with increasing overlap, between "self" and "dance group" correspond to a seven-point Likert scale, with higher scores indicating greater group closeness (Aron, Aron & Smollan, 1992).

Collective efficacy

This was measured using a one-item measure utilised in numerous studies (Cruwys et al., 2020). It was adapted to ask, "Together with other dance group members, we have been able to accomplish new dance skills and learn new choreography". It was measured on seven-point Likert scale, ranging from "1=strongly disagree" to "7=strongly agree". With higher rating indicating higher collective efficacy.

Group support

This was measured using a one-item question that was created for this study and included the statement "I feel supported by the dance group". It was measured on seven-point Likert scale, ranging from "1=strongly disagree" to "7=strongly agree". With higher rating indicating higher group support.

Group identity

This was measured using the four-item social identity scale (FISI) (Doosje, Ellemers & Spears, 1995). It was adapted to include statements such as i) "I identify with the dance group", ii) "I feel committed to the dance group", iii) "I am glad to be in the dance group", and iv) "Being in the dance group is an important part of how I see myself". It was measured on seven-point Likert scale, ranging from "1=strongly disagree" to "7=strongly agree". The items are averaged to yield a score from 1 to 7, with scores above 4 (the neutral point) indicating group identification, with higher rating indicating higher group identity.

Sensitivity analyses

For **anxiety**, there was evidence of differences across the eight weeks, $F(2, 30) = 6.15$, p=.01. For **loneliness**, there was evidence of differences across the eight weeks, $F(2, 30) = 4.45$, p=.03. For **depression**, there was evidence of differences across the eight weeks, $F(2, 30) = 4.12$, p=.03. For **wellbeing**, there was no evidence of differences across the eight weeks, $F(2, 30) = 1.48$, p=.246. For **self-esteem**, there was evidence of differences across the eight weeks, $F(2, 30) = 4.45$, p=.02. For **self-efficacy**, there was evidence of differences across the eight weeks, $F(2, 30) = 8.30$, p=.001. For **trust in the teacher**, there was no evidence of difference over the eight weeks, $F(2, 30) = 1.20$, p=.315. For **group closeness**, there was evidence of differences across the eight weeks, $F(2, 30) = 13.72$, p<.001. There was also no evidence of difference across week 4 and week 8 for **group support** $F(1, 13) = 0.17$, p=.686, **group identity** $F(1, 13) =.01$, p=.904, or **collective-efficacy** $F(1, 13) =.00$, p=1.00. Margins and pairwise comparisons for week 1, week 4, and week 8 using the full sample are presented in Table 1 below.

Table 2. Margins and pairwise comparisons for week 1, week 4, and week 8 (full sample).

	**Week 1**	**Week 4**	**Week 8**	**Week 1** vs. **Week 4**			**Week 1** vs. **Week 8**			**Week 4** vs. **Week 8**		
	Margins (predicted means)			Contrasts	95%CI	p-value	Contrasts	95%CI	p-value	Contrasts	95%CI	p-value
**Anxiety**	11.21	9.04	7.77	**-2.17**	**-4.05, -0.28**	**0.026**	**-3.44**	**-5.51, -1.37**	**0.002**	-1.27	-3.35, 0.80	0.219
**Loneliness**	6.20	5.42	5.21	**-0.78**	**-1.46, -0.10**	**0.026**	**-1.00**	**-1.75, -0.25**	**0.011**	-0.22	-0.97, 0.53	0.557
**Depression**	2.44	2.00	1.51	-0.44	-1.05, 0.16	0.145	**-0.94**	**-1.60, -0.27**	**0.008**	-0.49	-1.16, 0.18	0.143
**Wellbeing**	21.40	21.56	22.80	22.8	-1.44, 1.78	0.834	1.40	-0.37, 3.18	0.116	1.24	-0.53, 3.01	0.164
**Self-esteem**	3.55	3.88	4.39	0.33	-0.19, 0.86	0.205	**0.85**	**0.27, 1.42**	**0.006**	0.51	-0.07, 1.09	0.081
**Self-efficacy**	15.32	16.04	17.4	0.72	-0.22, 1.67	0.130	**2.08**	**1.03, 3.12**	**<.001**	**1.35**	**0.31, 2.40**	**0.013**
**Trust in teacher**	5.95	6.23	6.30	0.28	-0.19, 0.74	0.233	0.35	-0.16, 0.87	0.170	0.08	-0.44, 0.59	0.766
**Group closeness**	2.68	4.18	4.54	**1.50**	**0.77, 2.23**	**<.001**	**1.86**	**1.05, 2.66**	**<.001**	0.36	-0.45, 1.16	0.373
**Group support**		5.31	5.17							-0.14	-0.89, 0.60	0.686
**Group identity**		4.95	4.98							0.04	-0.59, 0.67	0.904
**Collective efficacy**		5.59	5.59							0.00	-0.45, 0.45	1.000

Predicted margins (means) of each measure at each timepoint are presented in column 1 for the full sample. Pairwise comparisons of these margins are presented in columns 2-4, where contrasts (differences) between margins across weeks are presented, alongside 95%CI (95% confidence intervals) and p-values. Estimates in bold indicate where there is evidence of change between weeks. Margins, contrasts, and 95%CI are rounded to 2 decimal places.

References

Anon (2008) *Short Warwick-Edinburgh Mental Wellbeing Scale (SWEMWBS)*. 2008. https://www.corc.uk.net/outcome-experience-measures/short-warwick-edinburgh-mental-wellbeing-scale-swemws/ [Accessed: 29 August 2022].

Aron, A., Aron, E.N. & Smollan, D. (1992) Inclusion of Other in the Self Scale and the Structure of Interpersonal Closeness. *Journal of Personality and Social Psychology*. 63 (4), 596–612. doi:10.1037/0022-3514.63.4.596.

Cruwys, T., Stewart, B., Buckley, L., Gumley, J. & Scholz, B. (2020) The recovery model in chronic mental health: A community-based investigation of social identity processes. *Psychiatry Research*. 291, 113241. doi:10.1016/J.PSYCHRES.2020.113241.

Doosje, B., Ellemers, N. & Spears, R. (1995) Perceived intragroup variability as a function of group status and identification. *Journal of Experimental Social Psychology*. 31 (5), 410–436. doi:10.1006/jesp.1995.1018.

Hughes, M.E., Waite, L.J., Hawkley, L.C. & Cacioppo, J.T. (2004) A Short Scale for Measuring Loneliness in Large Surveys: Results From Two Population-Based Studies. *Research on Aging*. 26 (6), 655–672. doi:10.1177/0164027504268574.

Kroenke, K., Spitzer, R.L. & Williams, J.B.W. (2003) The Patient Health Questionnaire-2: Validity of a Two-Item Depression Screener. *Medical Care*. 41 (11). https://journals.lww.com/lww-medicalcare/Fulltext/2003/11000/The_Patient_Health_Questionnaire_2__Validity_of_a.8.aspx.

Romppel, M., Herrmann-Lingen, C., Wachter, R., Edelmann, F., Düngen, H.D., Pieske, B. & Grande, G. (2013) A short form of the General Self-Efficacy Scale (GSE-6): Development, psychometric properties and validity in an intercultural non-clinical sample and a sample of patients at risk for heart failure. *GMS Psycho-Social-Medicine*. 10, 1–7. doi:10.3205/PSM000091.

Spitzer, R.L., Kroenke, K., Williams, J.B.W. & Löwe, B. (2006) A Brief Measure for Assessing Generalized Anxiety Disorder: The GAD-7. *Archives of Internal Medicine*. 166 (10), 1092–1097. doi:10.1001/archinte.166.10.1092.
